# P150: National action plan for the local production of alcohol-based hand rub for hand hygiene in Senegalese hospitals: situational analysis

**DOI:** 10.1186/2047-2994-2-S1-P150

**Published:** 2013-06-20

**Authors:** F Djiby, N Awa, K Rokhaya, N Babacar

**Affiliations:** 1 Fann Hospital, Dakar, Senegal

## Introduction

Hand hygiene is an important measure to reduce the risk of infections related to care proceedings. WHO proposes to use alcohol-based hand rubas a strategy for improving hand hygiene in health care settings.

## Objectives

As part oftheimplementation of this strategy, Senegal has initiated anational action planfor the installation ofmanufacturing unitsin hospital pharmaciesand national medicinessuppliers.

## Methods

one of the pilot hospital of the African Patient Safety Partnerships Program supported by the WHO for local production of alcohol-based hand rub is located in In Senegal. The experience of thishospitalis used toestablish apartnership between the Departmentof Health andIntrahealth, USAIDagencyinvolved inthe provision ofqualitycare.The objective ofthis partnership is to strengthen the capacity ofall pharmaciesin thelocal manufacturingof the product. To install these units the Minister of Health signed a circular allowing us to do a situational analysis of pharmacies. The objective was to assess the capabilities of each structure to house a manufacturing unit. The study was funded by Intrahealth. We visited 39 pharmacies.

## Results

In 35 pharmacies we found at least one pharmacist and a pharmacy technician in each structure. The premises were compliant production in 70% of cases. Only 12% of pharmacies had equipment.

## Conclusion

These results will be shared with the University Hospitals of Geneva.

## Disclosure of interest

None declared

